# Electronic Noses and Tongues in Wine Industry

**DOI:** 10.3389/fbioe.2016.00081

**Published:** 2016-10-25

**Authors:** María L. Rodríguez-Méndez, José A. De Saja, Rocio González-Antón, Celia García-Hernández, Cristina Medina-Plaza, Cristina García-Cabezón, Fernando Martín-Pedrosa

**Affiliations:** ^1^Group of Sensors, Escuela Ingenierías Industriales, Universidad de Valladolid, Valladolid, Spain

**Keywords:** electronic nose, electronic tongue, wine, multisensory, electronic panel

## Abstract

The quality of wines is usually evaluated by a sensory panel formed of trained experts or traditional chemical analysis. Over the last few decades, electronic noses (e-noses) and electronic tongues have been developed to determine the quality of foods and beverages. They consist of arrays of sensors with cross-sensitivity, combined with pattern recognition software, which provide a fingerprint of the samples that can be used to discriminate or classify the samples. This holistic approach is inspired by the method used in mammals to recognize food through their senses. They have been widely applied to the analysis of wines, including quality control, aging control, or the detection of fraudulence, among others. In this paper, the current status of research and development in the field of e-noses and tongues applied to the analysis of wines is reviewed. Their potential applications in the wine industry are described. The review ends with a final comment about expected future developments.

## Introduction

Wine is an alcoholic beverage, consisting of several hundred components in different ranges of concentrations. Among the techniques used to determine the quality of wines, the most important is sensory evaluation by trained experts, as it is directly related to the organoleptic characteristics (flavor, taste, and color) and quality of wines. Such an evaluation is carried out throughout the elaboration process from the analysis of the grapes to the evaluation of the final product. Only in a few cases are food and wine tasters used, under controlled conditions, as analytical instruments (Matthews et al., 1990). Such analytical panels are expensive and time consuming, and they are not always available. Wines are also characterized by classical chemical methods, such as gas and liquid chromatography or spectrophotometry, which can be used to obtain information about the presence or concentration of specific components. Chemical analysis is challenging due to the complexity of the mixture, and because minute variations in the concentration of certain compounds are responsible for distinct organoleptic characteristics. Moreover, interactions and synergies between groups of compounds often have a stronger influence on the organoleptic characteristics than individual components (Jackson, 2014).

The analysis of wines requires new technologies able to detect many different compounds simultaneously, providing global information about the sample instead of information about specific components.

Over the last few decades, a series of holistic methods have been developed to determine the quality of foods and beverages. In such methods, the instrumental signals (registered by FTIR spectra, mass spectrometry, NMR, chromatography, or signals provided by an array of sensors) are processed using a pattern recognition software to obtain a fingerprint of each sample, which can be used for discrimination or classification purposes. There is no need to separate the complex mixture into its individual components. Such holistic approaches are inspired by the method used in mammals to recognize food through their senses (Pioggia et al., 2008).

The most popular holistic methods are based on arrays of sensors, the so-called electronic noses (e-noses) and electronic tongues (e-tongues). Electronic senses have been used in the food industry for odor or taste analysis to evaluate the quality of a variety of products, including wine, fish, meat, beer, milk, water, etc. (Cosio et al., 2012; Wilson, 2013; Rodríguez-Méndez, 2016). The objective of this paper is to present the state of the art of e-noses and e-tongues and their application to the wine industry (Table 1).

**Table 1:** **E-noses and e-tongues and their applications in enology**.

Application		E-nose Sensor technology	Reference	E-tongue Sensor technology	Reference
Analysis of grapes and crushing		MOX	Prieto et al. (2011)	ISFET	Moreno i Codinachs et al. (2008)
				Voltammetric CPE	Prieto et al. (2011)
				Voltammetric Biosensor	Medina-Plaza et al. (2014a)
Improvement of maceration (flash release and micro-oxygenation)		MOX	Prieto et al. (2011)	Voltammetric CPE	Prieto et al. (2011)
Alcoholic fermentation		CP	Pinheiro et al. (2002)	Potentiometric	Buratti et al. (2011)
		MOX	Lozano et al. (2014)		
		MOX	Peris and Escuder-Gilabert (2013)		
		MOX + FTIR	Buratti et al. (2011)		
Oxygen level, phenolic content in red wines		MOX	Rodríguez-Méndez et al. (2014)	Voltammetric CPE	Rodríguez-Méndez et al. (2014)
Monitoring of aging in barrels		MOX	Wei et al. (2014)	Potentiometric	Rudnitskaya et al. (2007)
		MOX	Apetrei et al. (2012)	Potentiometric	Rudnitskaya et al. (2010)
		MOX	Lozano et al. (2008b)	Voltammetric	Parra et al. (2006b)
Aging with alternative methods		MOX	Santos et al. (2011)	Voltammetric CPE	Gay et al. (2010)
		MOX	Prieto et al. (2012)	Voltammetric CPE	Apetrei et al. (2007)
		MOX	Apetrei et al. (2007)		
Monitoring of aging in bottles: cork vs. polymeric stoppers		MOX	Prieto et al. (2011)	Voltammetric CPE	Rodríguez-Méndez et al. (2014)
Discrimination of organoleptic characteristics of the final product	Grape: variety/geographic origin/appellation	MOX	Villanueva et al. (2006)	ISFET	Artigas et al. (2003)
		MOX	Lozano et al. (2007a)	ISFET	Moreno i Codinachs et al. (2008)
		MOX	Aleixandre et al. (2008)		
		MOX	Lozano et al. (2007b)	ISFET	Gutiérrez et al. (2010)
				Voltammetric	Parra et al. (2004)
				Voltammetric	Rodríguez-Méndez et al. (2004)
				Voltammetric	Parra et al. (2006c)
				Voltammetric	Rodríguez-Méndez et al. (2008)
				Voltammetric	Cetó et al. (2014b)
				Voltammetric	Cetó et al. (2015)
				Biosensor	Cetó et al. (2014a)
				Biosensor	Medina-Plaza et al. (2014a)
	Geographical classification	MS	Cynkar et al. (2007)	Potentiometric	Legin et al. (1999)
		MOX	Berna et al. (2008)	Potentiometric	Legin et al. (2003)
		MOX	Buratti et al. (2004)	Potentiometric	Verrelli et al. (2007a)
		MOX	Rodríguez-Méndez et al. (2004)	Potentiometric	Rudnitskaya et al. (2010)
				Impedimetric	Riul et al. (2004)
		SAW	Beltrán et al. (2009)		
	Discrimination between wines with grape treatments	CP	Zoecklein et al. (2011)		
		CP	Devarajan et al. (2011)		
		QCM	López de Lerma et al. (2012)		
		QCM	Martín et al. (2008)		
Wine spoilage, off–flavors		MOX	Macías et al. (2013)	Potentiometric (all solid state)	Verrelli et al. (2007b)
		MOX	Santos et al. (2010)		
		MOX	Berna et al. (2008)	Potentiometric	Gil-Sánchez et al. (2011)
		MS	Cynkar et al. (2007)		
		–	Ragazzo-Sánchez et al. (2005)		
		MS	Martí et al. (2003)		
Detection of frauds and adulterations		QCM	Penza and Cassano (2004)	Potentiometric (all solid state)	Verrelli et al. (2007b)
				Voltammetric CPE	Parra et al. (2006c)
Assessment of chemical parameters		MOX	Macías et al. (2013)	Potentiometric	Legin et al. (2003)
		MOX	Santos et al. (2010)	Potentiometric	Kirsanov et al. (2012)
		MOX	Berna et al. (2008)	Potentiometric	Rudnitskaya et al. (2010)
		MS	Cynkar et al. (2007)	Potentiometric	Artigas et al. (2003)
					Gutiérrez et al. (2010)
				Potentiometric	Gutiérrez-Capitán et al. (2014)
				Voltammetric	Labrador et al. (2009)
				Voltammetric	Arrieta et al. (2003)
				Voltammetric	Apetrei et al. (2007)
				Voltammetric	Parra et al. (2004)
				Voltammetric	Prieto et al. (2011)
				Voltammetric	Cetó et al. (2012)
				Voltammetric	Cetó et al. (2014a)
				Voltammetric	García-Hernández et al. (2015)
				Biosensor	Gutiérrez-Capitán et al. (2014)
				Biosensor	Cetó et al. (2014b)
		QCM	Di Natale et al. (2004)	QCM	Di Natale et al. (2004)
		QCM	Lvova et al. (2006)	QCM	Lvova et al. (2006)
		QCM	Verrelli et al. (2007a)	QCM	Verrelli et al. (2007a)
		MOX	Rodríguez-Méndez et al. (2014)	Voltammetric CPE	Rodríguez-Méndez et al. (2014)
Correlations with human perceptions		MOX	Lozano et al. (2005)	Potentiometric	Di Natale et al. (2004)
		MOX	Lozano et al. (2006)	Potentiometric	Kirsanov et al. (2012)
		MOX	Lozano et al. (2007a)	Potentiometric	Legin et al. (2003)
		MOX	Santos et al. (2010)	Amperometric	Buratti et al. (2007)
		MOX	Arroyo et al. (2009)	Voltammetric	Cetó et al. (2015)
		QCM	Di Natale et al. (2000)	Voltammetric	Gay et al. (2010)

## Electronic Noses and Tongues

### Multisensor Systems Used in Electronic Noses

The *e-nose* has been defined as “*an instrument, which comprises an array of electronic chemical sensors with partial specificity and appropriate pattern-recognition system, capable of recognizing simple or complex odors*.” (Gardner and Bartlett, 1994; Rock et al., 2008).

Their purpose is to analyze aroma profiles by registering signals produced by the mixture of gases (as the human nose does), and then comparing the pattern of responses produced by different samples. They have been widely applied to the analysis of wines, including quality control, aging control, and the detection of fraudulence, among others (Rodríguez-Méndez, 2016). However, the analysis of wines is a not completely solved problem, due to the complexity of the samples, the minute differences in composition between different wines, and the presence of water and ethanol, which cause interference in the sensor responses (Horrillo et al., 2007). For this reason, intensive research is being dedicated to developing arrays of sensors with improved sensitivity and reproducibility.

Resistive sensors based on doped Metal Oxides (MOX) and MOSFETs are the most popular sensing units used in arrays dedicated to the analysis of wines (Santos et al., 2004; Smyth and Cozzolino, 2013). In spite of their high sensitivity to water, conducting polymers (CP) have also been successfully used in resistive sensors dedicated to analyzing wines (Pinheiro et al., 2005; Zoecklein et al., 2011). Arrays of optical sensors (Elosua et al., 2012), quartz microbalance (QMB) sensors (Di Natale et al., 2004; Zampetti et al., 2008), and surface acoustic wave sensors (SAW) (García et al., 2006) have also been successfully used for wine analysis.

Due to the high sensitivity of resistive sensors to water and ethanol, sampling techniques must be used not only to collect a representative headspace of the sample but also to eliminate, or at least to decrease, the concentration of water and ethanol in the volatile mixture (Lozano et al., 2007a). The sampling techniques commonly used in e-noses dedicated to wines include: Static Headspace (SH) (Penza and Cassano, 2004; Cozzolino et al., 2008), Purge and Trap (P&T) (Santos et al., 2004), and Solid Phase Micro Extraction (SPME) (Guadarrama et al., 2001; Villanueva et al., 2006; Lozano et al., 2008a). In SPME, absorbent resins (with low affinity toward water and ethanol) are used to collect the volatiles present in the headspace of the sample. Once adsorbed, volatiles are released by applying a temperature program and injecting them in the sensor chamber. Nowadays, SPME is the most popular method used in e-noses due to its high efficiency in eliminating water and ethanol.

### Multisensor Systems Used in Electronic Tongues (E-Tongues)

The success of e-noses led to the development of multisensor systems dedicated to the analysis of liquids. Such instruments were called taste sensors or e-tongues. According to the IUPAC, “*An electronic tongue is a multisensor system, which consists of a number of low-selective sensors and cross-sensitivity to different species in solution, and an appropriate method of pattern recognition and/or multivariate calibration for data processing*” (Vlasov et al., 2005; Lvova et al., 2013; Tahara and Toko, 2013; Sliwinska et al., 2014).

E-tongues can use arrays of sensors based on a variety of transduction principles (mass, optical, or electrochemical), but electrochemical sensors (potentiometric, amperometric, voltammetric, or impedimetric sensors) are the most widely used to design e-tongues (Del Valle, 2010; Kimmel et al., 2012).

In arrays of potentiometric sensors, the potential created across a selective membrane prepared from different materials (lipidic, polymeric, etc) is measured. This potential created at the interphase depends on the interaction electrode/solution, which in turn depends on the chemical compounds present in the analyzed solution (Ciosek and Wróblewski, 2011). Arrays of miniaturized Ion Selective Field Effect Transistors (ISFETS), micro Ion Selective Electrodes (μISE), or Light Addressable Potentiometric Sensors (LAPS) have also been developed using silicon planar technology (Bratov et al., 2010). Potentiometric multisensor systems have been successfully used to evaluate the organoleptic properties of wines (Zeravik et al., 2009; Rodríguez-Méndez, 2016).

Multisensor systems based on impedimetric sensors modified with organic materials (CPs, perylenes, phthalocyanines or carbon nanotubes) have also been successfully used to analyze wines (Volpati et al., 2012).

In amperometric/voltammetric sensors, a bias voltage is applied and compounds with redox activity are oxidized or reduced at a characteristic voltage (Winquist, 2008). Multisensor systems, based on amperometric or voltammetric sensors, are gaining interest in the wine sector because they can detect redox species, such as phenols (Makhotkina and Kilmartin, 2010). Experimental conditions can be tuned by applying different excitation functions (e.g., cyclic voltammetry, pulse voltammetry, or square wave voltammetry) or by establishing specific working ranges (Parra et al., 2004; Scampicchio et al., 2008; Winquist, 2008; Rodríguez-Méndez, 2016). In addition, they can be prepared using a variety of chemical modifiers with electrocatalytic properties (phthalocyanines, CPs, nanoparticles, or CNTs among others), thus improving the sensitivity (Parra et al., 2006a). Simultaneously, the interactions between the electrode and the solution greatly improve the selectivity of the electrodes. Such interactions include, among others: (i) the oxidant or reducing character of the solution that can modify the oxidation potential of the electrode material; (ii) the electrocatalytic activity of the electrode material that can facilitate the oxidation of the compounds dissolved in the test solution; (iii) the acid or basic character of the solution can protonate/deprotonate the electrode; and (iv) the nature and concentration of ions present in the solution that diffuse inside the sensing layer to maintain the electroneutrality (Rodríguez-Méndez et al., 2008).

Multitransduction systems combining different types of measurement are very popular in e-tongues (Lvova et al., 2015).

Finally, one of the most recent strategies in the field of e-tongues is to include biosensors with specificity toward certain compounds in the array. These systems are called bioelectronic tongues and are gaining interest in the wine industry as they provide global information about the wine (as classical arrays do) while simultaneously offering data about particular compounds, thanks to the specificity of the biosensors (Zeravik et al., 2009; Toko, 2013). For instance, biosensors containing phenol-oxydases (e.g., tyrosinase, laccase, or peroxidase) are used to detect phenols (Cetó et al., 2014a), while glucose oxidase or fructose dehydrogenase are used for the detection of sugars (Gutiérrez-Capitán et al., 2014; Medina-Plaza et al., 2014a,b). However, when designing arrays of sensors containing biosensors, it is necessary to keep in mind that each enzyme needs specific conditions to be functional. This means that each biosensor will need specific immobilization conditions and appropriate electron mediators.

### Multivariate Data Treatment

The combination of responses generated by the array of sensors is not specific for a certain compound, but may be related to certain characteristics of the samples by means of pattern recognition techniques. Four sequential steps are followed: signal pre-processing, dimensionality reduction, prediction, and validation (Beebe et al., 1998).

The signal pre-processing includes drift compensation, scaling, and the extraction of representative parameters. The dimensionality reduction step projects the initial feature onto a lower dimensional space (Prieto et al., 2012). This is usually carried out by means of a non-supervised technique, such as principal component analysis (PCA). PCA can be used to discriminate between samples with different organoleptic characteristics. The resulting low-dimensional feature vector is used to solve prediction problems, such as classification or regression.

**Figure 1:**
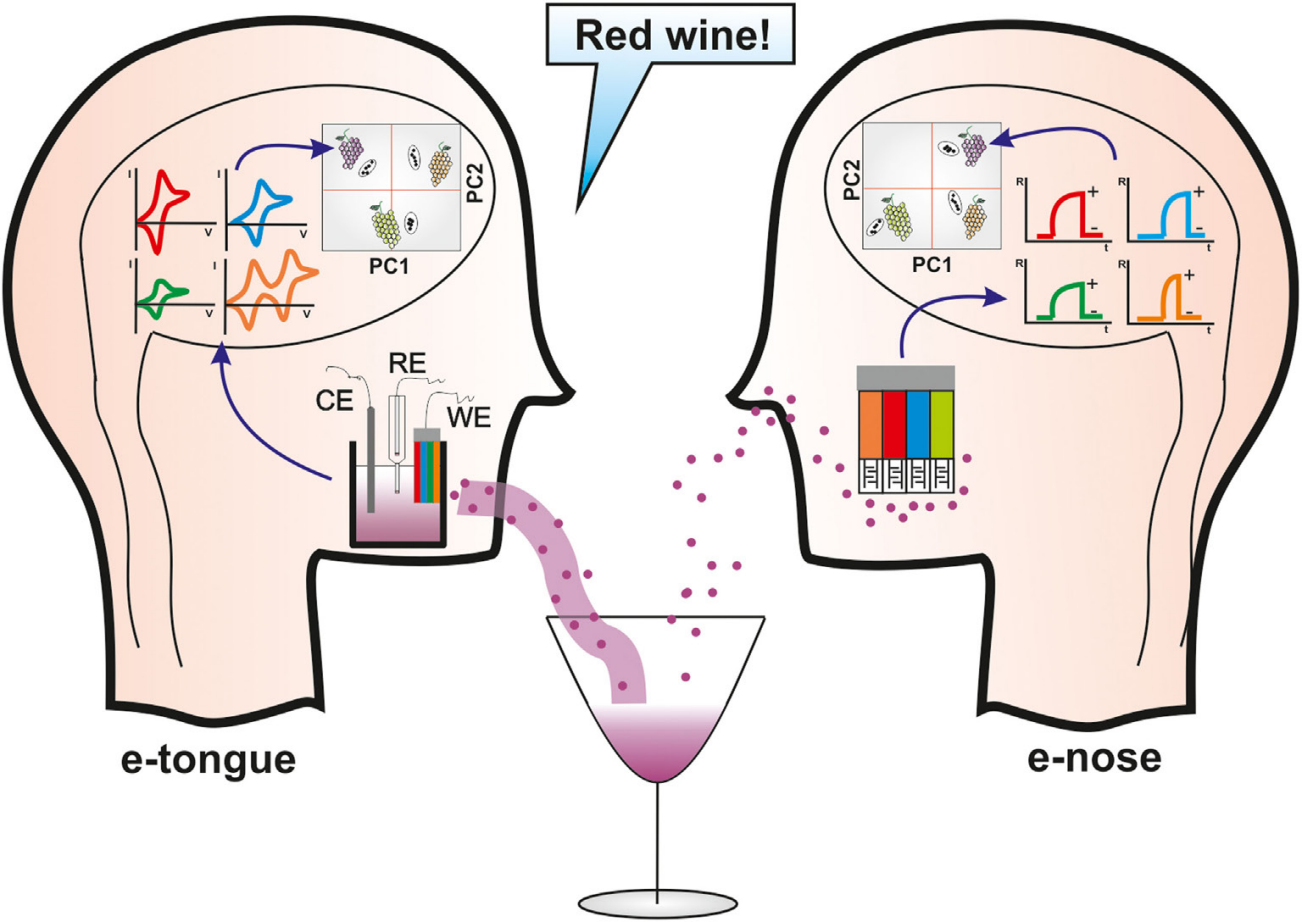
**Scheme of the working principle of e-tongues and e-noses**.

Classification tasks address the problem of identifying an unknown sample and assigning it to a certain set of previously learned categorized samples. Typical classification models are linear discriminant analysis (LDA), Soft Independent Modeling of Class Analogy (SIMCA), Support Vector Machines (SVM), or Artificial Neural Networks (ANN). In regression tasks, the goal is to establish a predictive model using a set of independent variables (e.g., sensor responses) and a second set of variables that are the properties of the sample analyzed (e.g., concentration, quality). The regression model is usually carried out using partial least square (PLS) (Kirsanov et al., 2012). The scheme of the working principles of e-noses and e-tongues is shown in Figure 1.

## E-Noses and E-Tongues in the Wine Industry

The organoleptic characteristics of wines depend on their chemical composition, which in turn depends on the variety of grape, the method used to crush the grapes, and the reactions occurring during vinification or during wine aging in barrels and bottles. E-noses and e-tongues have been applied to the quality control of wines at different stages of production, from the evaluation of the quality of grapes to the analysis of the quality and organoleptic properties of the bottled product (Smyth and Cozzolino, 2013; Loutfi et al., 2015; Rodríguez-Méndez, 2016) (Table 1).

### Assessment of the Quality of Grapes and Crushing

In enology, the ripeness and quality of the grapes is established on the basis of their external appearance or the taste of the grape juices. The analysis of the sugar and phenolic content is also a common practice to establish the quality.

E-tongues based on ISFETS (Moreno i Codinachs et al., 2008), or on voltammetric biosensors (Medina-Plaza et al., 2014a), have been used to discriminate between grapes according to the variety or vintage of the grape. Biosensors have established excellent correlations between the sugar and the phenolic content of mature grapes.

After crushing, musts and skins are left in contact for a period of time in order to increase the concentration of phenols in the wine. Several techniques, such as Flash release or micro-oxygenation, can be used to improve the extraction of phenols. An electronic panel, a combined system using an E-Nose, an e-tongue, and color measurements, has proven itself capable of detecting the increase in the phenol concentration (Prieto et al., 2011).

### Monitoring the Fermentation

The fermentation of fresh grape juice or must transforms sugars into alcohol, producing wine. In white wines, only one fermentation (the alcoholic fermentation producing ethanol) is needed. In red wines, the alcoholic fermentation is followed by the malolactic fermentation, where malic acid is transformed into lactic acid. Fermentations are usually controlled by measuring physical parameters (temperature and density) (Peris and Escuder-Gilabert, 2013). During malolactic fermentation, malic acid concentration is also monitored periodically. However, the control by means of chemical sensors is quite difficult, because fermentation is a turbulent process that produces changes in temperature that affect the performance of the sensors. For this reason, only a few papers have described the use of e-noses or e-tongues to monitor wine fermentation (Santos et al., 2015). However, a commercial e-nose based on CPs has been successfully used to monitor, on-line, the volatiles evolved during fermentation (Pinheiro et al., 2002). Using infrared spectroscopy, combined with an e-nose and e-tongue, the kinetics of the fermentation process of eight musts has been analyzed and acceptable correlations with sugar consumption and alcohol production have been found (Buratti et al., 2011).

It has been demonstrated that e-noses can be installed in cellars to monitor, on-line, the changes occurring in tanks using a sampling method that extracts the volatiles from the tanks where the wine is stored (Lozano et al., 2014).

### Monitoring the Aging of Wines in Oak Barrels

In order to improve the organoleptic characteristics, red wines are aged in oak barrels before bottling. In many regions (or appellations), wines are classified according to the length of the aging in barrels. During the aging process, volatiles are released from lees. In addition, minute amounts of oxygen are diffused into the barrel through the pores in the wood. The selection of the type of wood is important because oaks from different geographic origins or with different degrees of toasting can induce different flavors. The oxidative aging in barrels can be followed by an aging in bottles to further improve the organoleptic characteristics.

E-noses and e-tongues can be used to monitor or recognize the method of aging. For instance, e-noses based on MOX sensors can discriminate between red wines vinified under the same conditions but aged in oak barrels of different origins. Moreover, the measure of the changes in the volatile composition, measured periodically, can be a valuable tool to monitor aging (Lozano et al., 2008b; Apetrei et al., 2012; Wei et al., 2014). A potentiometric e-tongue has been able to classify Port wines of different ages (from 2 to 70 years) (Rudnitskaya et al., 2007) and Madeira wines produced from different varieties of grape and length of aging (Rudnitskaya et al., 2010). Similarly, a voltammetric e-tongue has been successfully applied to discriminate between red wines aged in oak barrels of different origin (French, Lithuanian, or American) with increasing toasting levels and to follow the changes experienced by wines after 3 and 6 months of aging (Parra et al., 2006b).

In alternative aging methods, wines are matured by soaking pieces of wood of different sizes (chips or staves) in micro-oxygenated stainless steel tanks. These methods avoid the use of expensive oak barrels and reduce the length of time required for aging. Such practices are legal in many countries, but their use must be indicated on the label. E-noses and e-tongues can be useful to detect the use of alternative aging methods (Apetrei et al., 2007; Santos et al., 2011; Prieto et al., 2012). In particular, a voltammetric e-tongue was able to detect the use of alternative methods and to discriminate between the effect of the size and type of the pieces of wood. Once wines were bottled, the differences could not be noticed (Apetrei et al., 2007; Gay et al., 2010). However, the panel of experts showed similar capabilities.

### Bottling

Natural cork stoppers are traditionally used in high-quality wines. Due to their porous nature, natural cork allows minute amounts of oxygen to diffuse into the wine. On the other hand, polymeric stoppers can be designed to have a certain porosity able to deliver controlled and reproducible amounts of oxygen into bottles. Recently, the effect of this nano-oxygenation has been successfully analyzed using a combined system of an e-nose formed by MOX sensors and an e-tongue formed by voltammetric sensors (Prieto et al., 2011). It was demonstrated that the e-tongue is more sensitive to the diffused oxygen than the e-nose. The combination of both techniques could detect not only the oxygen content, but also the polyphenolic level, which is related to the oxidation state of wines, which in turn is directly related to the organoleptic characteristics (Rodríguez-Méndez et al., 2014).

### Evaluation of the Organoleptic Characteristics of the Final Product

One of the main applications of e-noses and e-tongues in the enological sector is the evaluation of the quality of the final product. This includes the analysis of changes that occur over time (improving the bouquet), and also the detection of undesired changes due to cork damage or inappropriate storage.

E-noses and e-tongues can also be a valuable tool for controlling organisms in order to detect frauds that try to mislead the consumer, giving wrong information about the type of grape, geographical origin, etc.

The grape variety from which a wine is produced is responsible for the main organoleptic characteristics of the final product. In addition, the variability in weather conditions, or the high number of viticulture techniques and manipulations that can be applied during vinification and aging (such as the addition of yeasts, control of temperature, coupages, type of barrel, and so on), are the reason for the large number of wines with their own distinct flavors and characteristics. Finally, the change in the organoleptic characteristics, as aging continues in the bottle, improves the bouquet, but too long or inappropriate storage conditions can spoil the wine.

MOX based e-noses can easily discriminate distinctive varietal flavors found in wines elaborated from different grape varieties (Villanueva et al., 2006; Lozano et al., 2007a,b; Aleixandre et al., 2008), or grapes from different geographic origins or appellations (Buratti et al., 2004; Rodríguez-Méndez et al., 2004; Berna et al., 2008; Beltrán et al., 2009). Mass e-noses (using mass spectroscopy combined with chemometrics), or arrays of SAW sensors, have been successfully used to discriminate geographical origin (Cynkar et al., 2007; Beltrán et al., 2009).

Amperometric or voltammetric detection combined with an e-nose could classify Italian wines with different appellations (Buratti et al., 2004; Rodríguez-Méndez et al., 2004).

The changes in the mixture of volatiles occurring when grapes are submitted to different pre-harvest or post-bloom treatments can also be detected using e-nose systems (CP and surface acoustic wave-based) (Martín et al., 2008; Zoecklein et al., 2011). Other important aspects in viticulture, such as the effect of the canopy side (Devarajan et al., 2011), or of sun-drying and dehydration (López de Lerma et al., 2012), in the organoleptic characteristics of the final wine have also been evaluated with e-noses.

Regarding e-tongues, arrays of potentiometric chemical sensors have been extensively used to discriminate wines from the same appellation and vintage, but from different vineyards (Legin et al., 1999, 2003). Arrays of all-solid-state potentiometric sensors discriminated between Italian white wines of the Verdicchio appellation (Verrelli et al., 2007a). In Madeira wines, it was found that only the effect of age was significant for the electronic-tongue (Rudnitskaya et al., 2010). ISFETs-based arrays can also distinguish between wines elaborated from different grape types and vintage (Artigas et al., 2003; Moreno i Codinachs et al., 2008). Hybrid systems combining different types of sensors (ISFETs, conductivity, redox potential and amperometric) can also characterize and classify monovarietal white wines according to the grape variety and geographical origin (Gutiérrez et al., 2010).

Voltammetric sensors, chemically modified with electrocatalytic materials, have been widely used to discriminate wines from different Spanish regions or appellations, types of grape and vintage, and have been able to monitor the changes in wine occurring during aging in barrels and bottles. Worth noting is the high number of different chemical modifiers used in voltammetric arrays (phthalocyanines, CPs, CNTs, or NPs), which has led to a variety of arrays with different specifications (Parra et al., 2004, 2006c; Rodríguez-Méndez et al., 2004, 2008; Cetó et al., 2014a,b).

Impedimetric gold interdigitated sensors, modified with CPs/lipids, can also correctly distinguish wines, according to the vintage or vineyard (Riul et al., 2004).

As stated in previous paragraphs, introducing biosensors in the array can be advantageous, as they can introduce a certain selectivity toward specific compounds. In the case of the wine industry, the most common strategy is to use enzymes able to detect phenols or sugars, as these families of compounds have a great influence on the quality and organoleptic characteristics of wines. The specificity toward phenols is achieved by means of Tyrosinase or Lacasse, two phenol-oxydases that can be immobilized in graphite-epoxy electrodes (Cetó et al., 2014a), in carbon paste electrodes (CPEs), or in thin films (Medina-Plaza et al., 2014a). Glucose oxidase, or fructose dehydrogenase, are two examples of enzymes that can be introduced in arrays using similar techniques.

### Detection of Spoilage and Off-Odors

The quality of a wine can be altered by the presence of certain unwanted chemical compounds formed during fermentation or storage. The early detection of these off-odors is crucial to be able to undertake remedial actions that can correct the fault. Some important volatile compounds responsible for off-odors have been successfully detected using e-noses. They include acetic acid (Macías et al., 2013), ethyl acetate or sulfur compounds (Santos et al., 2010), or 4-ethylphenol and 4-ethylguaiacol caused by brettanomyces yeasts (Cynkar et al., 2007; Berna et al., 2008). Off-odors associated with damaged corks are due to the presence of chlorinated compounds, such as 2,4,6-Trichloroanisole. Such compounds can be detected by the human nose even if they are in extremely small concentrations. For this reason, classical arrays of sensors cannot be used for this purpose and must be associated with chromatography (Ragazzo-Sánchez et al., 2005). Mass spectrometry coupled to data treatment can be used for this purpose (Martí et al., 2003).

Spoilage due to the presence of different compounds in wines can also be detected using e-tongues. For instance, an e-tongue composed of “all-solid-state” potentiometric sensors was able to monitor the levels of acetic acid in white wines (Verrelli et al., 2007b). An e-nose combined with an array of potentiometric sensors has been used to monitor the spoilage of wines that was also followed by the titrable (total) acidity (Gil-Sánchez et al., 2011).

### Detection of Frauds and Adulterations

Wine producers must follow the regulations indicated by their appellations and by national and international organisms. However, irregular practices (dilution with water, addition of substances, use of grapes from different regions, or using forbidden aging methods…) are carried out in order to decrease the costs. E-noses and e-tongues can be a valuable tool in detecting such practices. As already mentioned in the previous paragraphs, electronic systems can be used to discriminate wines elaborated using different grapes and techniques. This capability can be used for quality control in cellars, but also by official organisms to certify the authenticity of the product, complementing the results obtained by traditional analytical techniques.

E-noses can detect the adulteration of wines with methanol or ethanol (Penza and Cassano, 2004). Voltammetric e-tongues have been used to detect adulteration with ethanol and also the addition of other non-volatile adulterants (tartaric acid, tannic acid, SO_2_, acetic acid or sucrose, and ethanol) (Parra et al., 2006c). A miniaturized potentiometric e-tongue has been able to detect H_2_S, SO_2_, and acetic acid in artificial wines (Verrelli et al., 2007b).

### Assessment of Chemical Parameters

Due to their working principle, e-noses and e-tongues provide global information about a sample. This is why their main use is the discrimination or classification of the samples by means of chemometrics. Nevertheless, as the signals obtained from the sensors are the result of the interaction of the sensing layer with certain compounds or mixtures of compounds, correlations can be established between the responses of the sensors and the chemical composition of the sample. For this reason, chemometric tools, such as PLS regression can be used to establish mathematical models between the output of the array of sensors and the chemical data obtained by classical methods. In this way, information about particular parameters can be obtained (Oliveri et al., 2010; Kirsanov et al., 2012). Moreover, once calibrated, e-noses and e-tongues can predict the concentration of many chemical parameters simultaneously.

As stated before, e-noses based on MOX sensors or MS have been successfully used to quantify several volatile compounds, including acetic acid (Macías et al., 2013), ethyl acetate, eugenol, ethyl octanoate, sulfur compounds (Santos et al., 2010), or guayacol (Cynkar et al., 2007; Berna et al., 2008).

Total polyphenols, sugar content, total and volatile acidity, pH, etc., can be predicted from e-tongue responses. Potentiometric, ISFETs, amperometric, voltammetric, and impedimetric sensors have been used for this task. For instance, potentiometric e-tongues have been able to quantify total and volatile acidity, pH, ethanol content, tartaric acid, sulfur dioxide, total polyphenols, and glycerol, with a precision of within 12% (Legin et al., 2003). In another instrument, free and total sulfur dioxide, total acidity, ethanol, pH, and some phenolics have been accurately detected (Kirsanov et al., 2012). Multisensor systems based on the combination of potentiometric sensors prepared from glass membranes and plasticized PVC can be used to detect tartaric, citric, formic, vanillic and sinapic acids, catechin, vanillin, and *trans*-resveratrol (Rudnitskaya et al., 2010).

Arrays of miniaturized FETs and ISFETs can also be used to measure pH, calcium, and potassium, necessary to control the tartaric stabilization of wines (Artigas et al., 2003). Electrochemical microsensors have also been combined with other measuring systems to assess the concentration of chemical parameters in wines (Gutiérrez et al., 2010; Gutiérrez-Capitán et al., 2014).

Voltammetric and amperometric sensors are particularly interesting for the analysis of wines because they can detect compounds with redox activity, such as polyphenols, sugars, or sulfites. Examples found in the literature include e-tongues based on simple metallic electrodes used to detect bisulfites (Labrador et al., 2009) and e-tongues based on electrodes chemically modified with phthalocyanines (Arrieta et al., 2003) that correlate well with up to 24 chemical parameters, including excellent correlations with the polyphenolic content (measured as total polyphenol index) acidity (measured as pH or total acidity) (Parra et al., 2004; Apetrei et al., 2007; Prieto et al., 2011), oxygen concentration, or antioxidant capacity (Rodríguez-Méndez et al., 2014).

An array of Electrochemical Quartz Crystal Microbalance (EQCM) sensors chemically modified with phthalocyanines has demonstrated its capability for detecting antioxidants and sugars in musts using voltammetry and mass measurements simultaneously (García-Hernández et al., 2015).

The antioxidant capacity of wines has also been evaluated using voltammetric sensors modified with other electrocatalytic modifiers, such as nanoparticles (Cetó et al., 2012, 2014a), or biosensors modified with phenol-oxidases (to detect phenols) or glucose oxidases to detect sugars (Cetó et al., 2014b; Gutiérrez-Capitán et al., 2014).

Arrays of gas and liquid sensors based on porphyrins used in a combined manner were able to quantify sugar, acidity, pH, tartaric, malic and lactic acids, polyphenols, antocyans and ions (Ca, Mg, and K) (Di Natale et al., 2004), alcoholic content (Lvova et al., 2006), and SO2, l-Malic acid, and total phenols index (Verrelli et al., 2007a).

The correlation coefficients and the limits of detection can be drastically improved when introducing biosensors into the array (Cetó et al., 2014a; Medina-Plaza et al., 2014a).

### Correlations with Human Perceptions

The final quality of wines is related to human perceptions. It is usually evaluated by a panel of trained experts who are able to appreciate different attributes and give a score. Because e-noses and e-tongues are inspired by human senses, one could expect that their outputs should be related to the scores given by a panel of experts. Unfortunately, the correlations with human perceptions are not easy to establish due to several factors. For instance, unlike the human systems, e-noses and e-tongues detect chemical compounds, whether they are species that have an odor or are odorless (taste or tasteless). In many cases, the intensity of the perception is not directly correlated with the concentration of a certain compound. Quite often, compounds in a mixture have a synergistic effect and the resulting aroma or taste is not the addition of the smell or taste of the individual components. Finally, human taste also perceives the feel in the mouth (astringency, heat, viscosity, etc.) or flavors that contribute to the perception as well. Thus, a complete description of the organoleptic properties is not possible from an e-tongue point of view.

Predicting the organoleptic properties of wines using electronic senses is particularly difficult because wines are extremely complex mixtures and because panels of experts evaluate how pleasant a perception is. However, pleasure and perceptions are not linearly related to a compound or a family of compounds.

In spite of the difficulties, using the appropriate array of sensors and PLS regression, it is possible to establish good correlations with sensory scores or with specific odors or flavors.

An e-nose combining 16 tin oxide thin film-based sensors has been used to recognize 29 typical aromas in white wine grouped into different families (floral, fruity, microbiological, herbaceous, and chemical) (Lozano et al., 2005), or to detect the main aromas usually present in red and white wines (Lozano et al., 2006; Santos et al., 2010). However, in general, panels of experts are more efficient at identifying aromas than e-noses (Arroyo et al., 2009). It has been reported that results provided by an e-nose corresponded better to the scores given by the panel of experts than the predictions made by chromatography (Lozano et al., 2007a). Good correlations have also been found with sensory scores and the outputs of arrays of potentiometric (Legin et al., 2003; Di Natale et al., 2004; Kirsanov et al., 2012), amperometric (Buratti et al., 2007), or voltammetric sensors (Gay et al., 2010; Cetó et al., 2015).

## Combination of E-Noses and E-Tongues

Flavor perception is based on both taste and aroma and can be analyzed using a combination of an e-nose and an e-tongue. The simultaneous use of both systems increases the amount of information extracted from a certain sample, enhancing the prediction capabilities. Results can be used to assess the presence of certain compounds in wines, or to classify samples in good agreement with a panel of experts. For instance, an e-nose and an e-tongue based on porphyrins could be used to quantify sugar, acidity, pH, tartaric, malic and lactic acids, polyphenols, antocyans, and ions (Ca, Mg, and K) in wines. Results demonstrate the capability of such systems to be trained according to data given by panels of tasters (Di Natale et al., 2000, 2004).

Combined e-noses and e-tongues have also been used to analyze the influence of corks in parameters related to the oxygen (diffused oxygen and antioxidants) (Prieto et al., 2011). E-noses and e-tongues have also been combined with an electronic eye to form an electronic panel that was able to recognize organoleptic characteristics of wines prepared using the same methodology, but from different varieties of grapes (Rodríguez-Méndez et al., 2004).

## Conclusion and Future Trends

E-noses and e-tongues consist of a series of non-specific sensors with cross-sensitivity that respond to a wide variety of compounds. After an appropriate training, they can be applied in the wine industry to give qualitative information about the sample, predict the concentration of certain compounds or detect certain organoleptic characteristics. The complete organoleptic description is not possible, but e-noses and e-tongues provide objective responses independent of physiological conditions or personal preferences, and they do not show fatigue (as human senses do). They have been shown to be a valuable tool for use in combination with classical analytical techniques to make key decisions regarding harvesting, crushing, fermentation, or aging. They could be used by enologists to control the quality of wines, but also by official organisms to detect frauds and illegal practices.

Some authors have expressed their criticisms because the main problem with e-noses and tongues is the lack of odor or taste sensors (Boeker, 2014). In any case, e-noses or e-tongues are able to detect chemical compounds and this is important for quality control purposes. Many groups are working on the development of new sensors with improved selectivity, and the performance of multisensor systems has improved greatly, particularly with the use of biosensors. Validation is also a problem in e-noses and e-tongues (Fonollosa et al., 2016). In the case of wines, the problem of weak validations is particularly important, because wines change with time, and quality depends on many odd conditions (i.e., weather). So, it is very difficult to have a large number of well characterized samples to carry out an appropriate training. The way to solve this problem is to construct international databases. In the field of e-tongues applied to wine, several laboratories are currently working on the construction of a database that will help to improve the quality of validations (Khaydukova et al., 2016).

Future strategies will also include the design of arrays formed by new materials with improved selectivity and sensitivity, in many cases linked to nanotechnology or with biosensors.

Efforts have to be made to introduce these instruments in cellars and in the list of recommended analytical tools established by the National and International Commissions.

## Author Contributions

MR-M wrote the paper. All authors listed have also made substantial, direct, and intellectual contribution to the work, and approved it for publication.

## Conflict of Interest Statement

The authors declare that the research was conducted in the absence of any commercial or financial relationships that could be construed as a potential conflict of interest.
